# Droplet Breakup Dynamics in Bi-Layer Bifurcating Microchannel

**DOI:** 10.3390/mi9020057

**Published:** 2018-01-31

**Authors:** Yong Ren, Kai Seng Koh, Maxine Yew, Jit Kai Chin, Yue Chan, Yuying Yan

**Affiliations:** 1 Department of Mechanical, Materials and Manufacturing Engineering, University of Nottingham Ningbo China, Ningbo 315100, China; maxine.yew@nottingham.edu.cn; 2 School of Engineering and Physical Sciences, Heriot-Watt University Malaysia, No. 1 Jalan Venna P5/2, Precinct 5, 62200 Putrajaya, Malaysia; k.koh@hw.ac.uk; 3 Department of Chemical Sciences, University of Huddersfield, Queensgate, Huddersfield HD1 3DH, UK; j.chin@hud.ac.uk; 4 Institute of Advanced Study, Shenzhen University, Nanshan District, Shenzhen 518060, China; unimelbat@hotmail.com; 5 Research Group of Fluids and Thermal Engineering, University of Nottingham Ningbo China, Ningbo 315100, China; 6 Research Group of Fluids and Thermal Engineering, Faculty of Engineering, University of Nottingham, Nottingham NG7 2RD, UK; yuying.yan@nottingham.ac.uk

**Keywords:** microfluidics, droplet fission, encapsulation, emulsions, breakup

## Abstract

Breakup of droplets at bi-layer bifurcating junction in polydimethylsiloxane (PDMS) microchannel has been investigated by experiments and numerical simulation. The pressure drop in bi-layer bifurcating channel was investigated and compared with single-layer bifurcating channel. Daughter droplet size variation generated in bi-layer bifurcating microchannel was analyzed. The correlation was proposed to predict the transition between breakup and non-breakup conditions of droplets in bi-layer bifurcating channel using a phase diagram. In the non-breakup regime, droplets exiting port can be switched via tuning flow resistance by controlling radius of curvature, and or channel height ratio. Compared with single-layer bifurcating junction, 3-D cutting in diagonal direction from bi-layer bifurcating junction induces asymmetric fission to form daughter droplets with distinct sizes while each size has good monodispersity. Lower pressure drop is required in the new microsystem. The understanding of the droplet fission in the novel microstructure will enable more versatile control over the emulsion formation, fission and sorting. The model system can be developed to investigate the encapsulation and release kinetics of emulsion templated particles such as drug encapsulated microcapsules as they flow through complex porous media structures, such as blood capillaries or the porous tissue structures, which feature with bifurcating junctions.

## 1. Introduction

Emulsions are ubiquitous in the daily life and have been extensively applied in food industry, cosmetics, pharmacy, and biochemical reactions [1,2,3]. Emulsions are formed in a mixture of two immiscible fluids, where the dispersed phase is suspended as droplets in the continuous phase. The droplets can be used as templates to synthesize substances such as microparticles or microcapsules [4,5], which lead to wide range of biomedical, chemical and industrial applications, including consumer and personal care products [6], ultrasound contrast agents [7], particle-based display [8,9], food additives [10], and photonic materials [11,12]. Microparticles and microcapsules can also be extensively used in energy-related applications such as energy storage [13], gas capture and sensing [14] as well as carbon dioxide capture and storage [15,16,17,18]. Emulsions are normally formed by intense shear or mechanical agitation in macroscopic systems. Nevertheless, the applications of the conventional emulsification methods are hindered because of drawbacks such as wide size distribution, low energy efficiency and poor control over encapsulation rate and release kinetics [19]. Microfluidic emulsification has demonstrated a high degree of control over the droplet formation, because the size, shape, and concentration of droplets can be finely tuned [20]. Droplets can be passively formed by fluid instabilities using coaxial [21], flow-focusing [22], T-junction [23], or step emulsification [20]. Large-scale complex biological and chemical assays demand generation of droplets with high throughput. This can be achieved by droplet fission, integration of various droplet formation mechanisms, or parallelization of multiple droplet microfluidic systems. In particular, droplet fission enables single droplets to be divided into two or multiple daughter droplets and it is therefore an indispensable operation for producing sample replicates, multiplexing of a large number of droplets, pairing and mixing individual droplets with different reagents [24]. Rapid and efficient mixing in daughter droplet with smaller volume leads to significant reduction of biochemical reaction time [25]. 

Droplets can be split in active or passive manners, while the passive fission is more widely applied mainly because of ease of implementation and low cost. Geometrically mediated breakup of droplets relates with the fluid flow resistance and geometries in respective channels of the microfluidic system. A variety of geometries have been demonstrated in passive fission where a droplet is split as it flows past T-junction [26], arbitrary angled-junction [27], obstacle [28], bifurcating junction [29], cross junction [30] or through a narrow constriction [31]. Droplets with the desired small volume can be achieved using cascading Y-shaped splitting array where large droplets undergo splitting for several times [32]. Droplet fission by bifurcating junction is normally implemented in 2-D manner using single-layer device [29,32]. The droplet splitting is closely related with the geometry of the tip of the bifurcating junction and the consistency of liquid flow. Sharp tip can attain high droplet splitting efficiency, and blunt tip is not favorable. Considerable surface roughness of microchannel creates inconsistent flow in axial direction and hence leads to pressure change in lateral direction across the channel, driving the droplets to be deviated from center of cross section of the channel. Consequently droplets may bypass the bifurcating junction and enter a side channel without undergoing fission. This requires fabrication with high resolution and high accuracy yet high cost to form sharp tip of the bifurcating junction and smooth channel surfaces. In our previous study, a novel 3-D bi-layer polydimethylsiloxane (PDMS) microchannel formed by bifurcating junction was proposed [33,34]. A numerical study demonstrates that compared with single-layer bifurcating junction, 3-D cutting in diagonal direction from bi-layer bifurcating junction leads to droplet fission in more effectively way, obviating the need of expensive fabrication approach to ensure the microchannel has exquisitely controlled surface roughness and extremely sharp tip of bifurcating junction. The droplet formation and fission in the microstructure using shear-thinning/Newtonian system was also investigated, and compared with Newtonian/Newtonian system [33]. A more complete and updated study of droplet generation in shear-thinning fluids can also be found in the work of Chiarello et al. [35].

Nevertheless, the droplet breakup dynamics in confined geometries such as the proposed structure has not been adequately studied, and it demands in-depth investigation. For example, pressure drop is the driving force of fluid flow in bifurcating microchannel for the formation and fission of droplets. High-pressure drops may lead to high energy consumption. The examination of pressure drop between single- layer and bi-layer bifurcating microchannel is thus necessary. The criterion based on Capillary number for the transition between the breakup and non-breakup regime has been investigated for single- layer bifurcating channel when the mother droplets interact with the tip of the bifurcation [36]. The validity of the understanding has not been adequately confirmed in bi-layer bifurcating channel. The bifurcating junction arranged in two separate layers also leads to asymmetric splitting of mother droplets, leading to formation of daughter droplets with distinct sizes [34], however the mechanism for forming daughter droplets with different sizes has not been clearly elucidated, and the effect to daughter droplet size variations due to Capillary number has not been investigated quantitatively by experiments. These examples attest to the need for a comprehensive understanding of the role of pressure drop and Capillary number associated with the droplet breakup dynamics in bi-layer bifurcating microchannel. 

In this study, the formation of single emulsion droplets at upstream T-junction and splitting of droplets at downstream bi-layer bifurcating junction have been investigated via numerical simulation and experiments. First, the pressure drop in bi-layer bifurcating channel was investigated and compared with counterpart in the form of single-layer. Subsequently, daughter droplet size variation was confirmed from experimental study of bi-layer bifurcating microchannel, hypothesis was made to relate the size variation with the different pressure distribution in upper and lower channels, and the hypothesis was verified. Analysis was performed to determine the daughter droplet volume ratio at various Capillary numbers in bi-layer bifurcating channel and compared with that in single-layer bifurcating channel. Finally, a correlation was proposed to predict the transition from breakup to non-breakup of droplets in bi-layer bifurcating channel. Understanding of the mechanism of droplet fission in the microstructure can lead to more versatile control over the emulsion formation and fission, and the release kinetics when the emulsion-templated microparticles are used for encapsulation and releasing application. The novel droplet fission approach provides insights for the design and fabrication of higher order emulsions with custom-made volume and shape, inspiring new biomedical and industrial applications based on microfluidic emulsification platform. 

## 2. Microchannel Design and Experimental Setup

**Figure 1:**
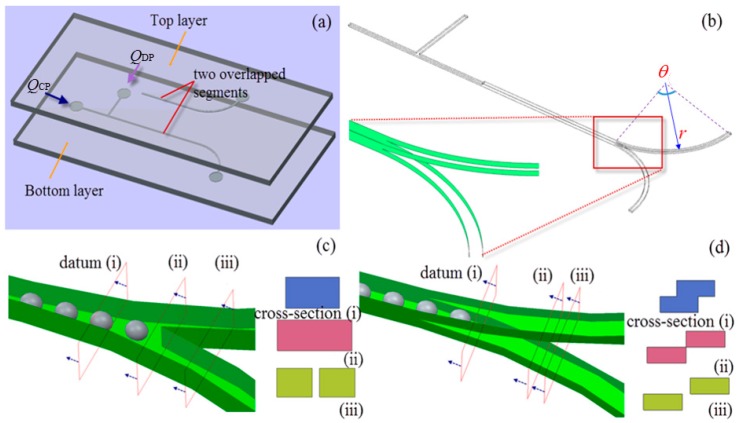
(**a**) Schematic of bi-layer bifurcating microchannel for droplet formation and fission; (**b**) enlarged view of 3-D bi-layer bifurcating junction; (**c**) layout and cross-sectional view of single-layer bifurcating junction; (**d**) layout and cross-sectional view of bi-layer bifurcating junction, the view direction is denoted by arrow.

Water-in-oil immiscible multiphase system is formed in a microdevice with a T-junction for droplet generation at upstream, where water is injected as the dispersed phase at a constant average flow rate *Q*_DP_, and oil is injected as the continuous phase at a constant average flow rate *Q*_CP_ (see Figure 1a). The subscripts DP and CP represent dispersed phase and continuous phase, respectively. The microdevice consists of two layers, each of which has patterned channels. An overlapped segment exists between channels in top and bottom layers to ensure good alignment, the design, as shown later, will also help to reduce total pressure drop. Two curved branch channels extending from the bifurcating junction lay on the top and bottom layer, respectively, forming a platform for droplet fission in confined bi-layer structure by shear stress exerted in 3-D manner (see Figure 1a,b). Each curved branch channel has rectangular cross-section with height of 100 µm and width of 250 µm, and the geometry is determined by radius of curvature, *r*, and included angle, θ (see Figure 1b). The layout and cross-sectional views of conventional single-layer bifurcating junction versus novel bi-layer bifurcating junction are compared in Figure 1c,d, illustrating that the cross-sectional area of bi-layer constriction keeps shrinking towards a singularity point which demarcates the onset of complete separation of two branch curved channels. Given the geometric variations, the droplet fission by bi-layer bifurcating junction will be investigated thoroughly to elucidate the droplet breakup mechanism in confined 3-D bi-layer bifurcating structure.

**Figure 2:**
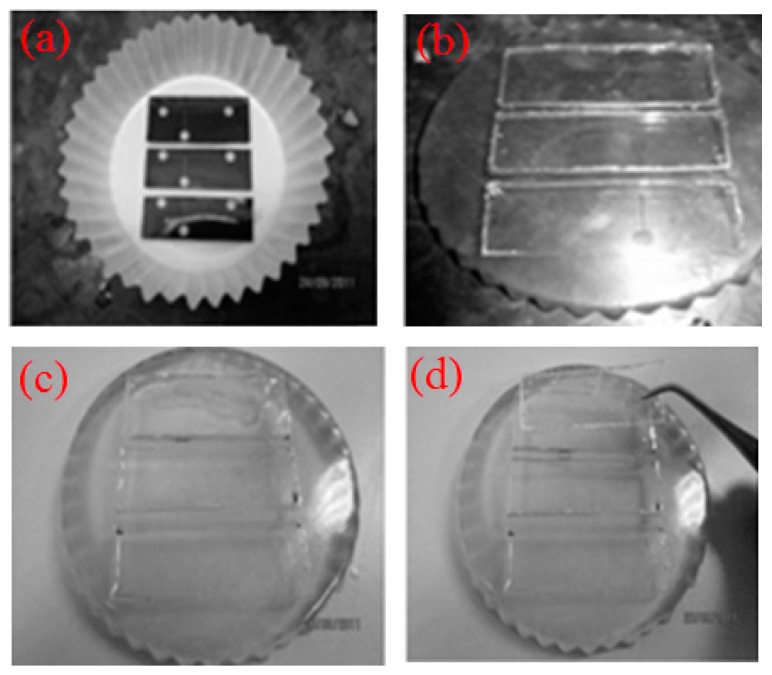
(**a**) The polydimethylsiloxane (PDMS) slab attached with patterned adhesive vinyl film was placed in cupcake; (**b**) the epoxy mold; (**c**) pouring PDMS mixture in the epoxy mold; (**d**) after curing, the PDMS layer was peeled from the mold.

**Figure 3:**
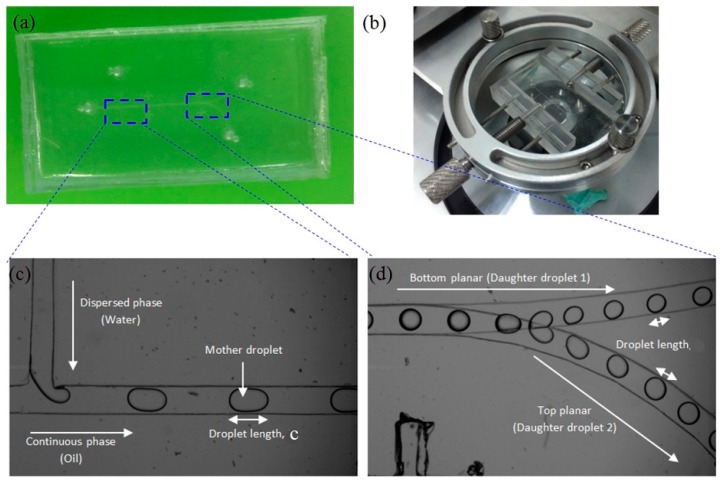
(**a**) Fabricated microdevice; (**b**) alignment system for partial curing control; (**c**) observed droplet formation at upstream; (**d**) droplet fission and formation of daughter droplets in upper and lower channels from two layers.

The device was fabricated using xurography approach [37]. First, the geometric pattern of microchannel was designed by CAD software (version 2016, Autodesk, Inc., San Rafael, CA, USA), and the pattern was sent to cutter plotter (CE6000-60, Graphtec Corporation, Tokyo, Japan) which has a blade to cut on the surface of adhesive vinyl film (Oracal, 651) with thickness of 100 μm. After the undesired part was peeled off, the film was attached to a blank PDMS slab, and then placed in a cupcake (see Figure 2a). The epoxy resin (Epo-Thin, Buehler, Illinois Tool Works Inc., Glenview, IL, USA) and hardener with weight ratio of 2:1were mixed and degassed, the epoxy mixture was poured into the cupcake and cured at 25 °C for 12 h to generate an inexpensive and durable epoxy mold for replica production by removing the blank PDMS slab. If size allowed, more than two epoxy molds can be fabricated from one cupcake (see Figure 2b). PDMS pre-polymer (Dow Corning, Sylgard 184, Midland, MI, USA) was mixed thoroughly with the curing agent at ratio of 10:1 (*w*/*w*). The mixture was degassed in a vacuum desiccator before it was poured into the epoxy mold and cured at room temperature for 24 h (see Figure 2c). The half-cured top and bottom PDMS layers were peeled from the respective epoxy mold with the aid of a blade (see Figure 2d). The top layer of PMDS channel was punched with 2-mm inlet and outlet ports. The two PDMS layers were aligned under the optical microscope with assistance of alignment rig to ensure the sealing at bifurcation (see Figure 3b). The bonding between the two PDMS layers was achieved using partial curing method [38] via thermally curing the aligned microfluidic device at 90 °C for 2 h. The final fabricated device is shown in Figure 3a. Scanning electron microscope (SEM) (Quanta400F, FEI, Thermo Fisher Scientific, Waltham, MA, USA) was used to measure the cross-sectional dimensions.

**Figure 4:**
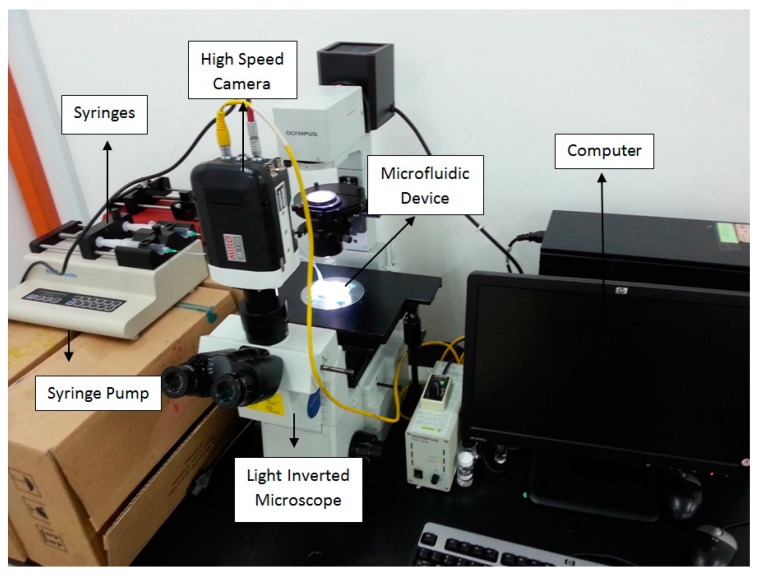
Experimental setup.

In the experiment, cooking oil served as the continuous fluid with viscosity of 0.0643 ± 0.0016 Pa·s measured by a viscometer (LVDV-II+P CP, Brookfield Engineering, Middleboro, MA, USA). Deionized (DI) water was used as the dispersed fluid. The interfacial tension of 14.26 mN/m between water and oil was measured by a goniometer (ramé-hard Instrumental Co., Succasunna, NJ, USA). The properties of the test fluids are shown in Table 1. The volumetric flow rates of continuous phase and dispersed phase were controlled by syringe pumps (KDS 210, KD Scientific Inc., Holliston, MA, USA), separately. The droplet formation and fission were recorded under a high speed camera (M110, Phantom, Vision Research Inc., Wayne, NJ, USA) mounted on top of an inverted microscope (IX51, Olympus, Tokyo, Japan). The software ImageJ (version 1.48v, National Institutes of Health (NIH), Bethesda, MD, USA) was applied for image analysis to evaluate droplet Feret’s diameter *D* which represents the longest distance between any two points on a selection boundary. In our measurement, it can be used to determine the maximum diameter of a droplet when the shape is not strictly spherical. The mother droplet was generated by T-junction to form water-in-oil emulsion (see Figure 3c), which travelled downstream and was split up bifurcating junction with formation of trains of daughter droplets in bottom and top-layer branch microchannel, respectively (see Figure 3d). The complete experimental setup is shown in Figure 4.

## 3. Numerical Model

A 3-D numerical model of the droplet formation and fission in the bi-layer bifurcating microchannel was established. The two-phase flow problem was solved using the VOF (Volume of Fluid) method by CFD software Ansys Fluent 15.0 (Ansys, Inc., Canonsburg, PA, USA). In this method, the interface between water and oil phase was tracked by solving the following convection equation [39,40]:

(1) $$\frac{\partial \alpha_{\mathrm{CP}}}{\partial t}+V\cdot \nabla \alpha_{\mathrm{CP}}=0$$

where *α* denotes volume fraction, *t* and *V* denote time and flow velocity respectively, and the subscript CP represents continuous phase. 

The governing equations for incompressible two-phase Newtonian fluids can be described by continuity equation (Equation (2)) and Navier–Stokes equation (Equation (3)), the cell-averaged density and dynamic viscosity can be obtained from Equations (4) and (5).

(2) $$\frac{\partial \rho }{\partial t}+\nabla \cdot \left(\rho V\right)=0$$

(3) $$\frac{\partial }{\partial t}\left(\rho V\right)+\nabla \cdot \left(\rho VV\right)=-\nabla P+\nabla \cdot \left[\eta \left(\nabla V+\nabla V^{T}\right)\right]+\frac{\rho \sigma \nabla \alpha_{\mathrm{CP}}}{\frac{1}{2}\left(\rho_{\mathrm{DP}}+\rho_{\mathrm{CP}}\right)}\nabla \cdot \frac{\nabla \alpha_{\mathrm{CP}}}{\left|\nabla \alpha_{\mathrm{CP}}\right|}$$

(4) $$\rho =\alpha_{\mathrm{CP}}\rho_{\mathrm{CP}}+\left(1-\alpha_{\mathrm{CP}}\right)\rho_{\mathrm{DP}}$$

(5) $$\eta =\alpha_{\mathrm{CP}}\eta_{\mathrm{CP}}+\left(1-\alpha_{\mathrm{CP}}\right)\eta_{\mathrm{DP}}$$

where *P* is the pressure, ρ is the volume averaged density, η is the volume averaged dynamic viscosity, and *σ* is the interfacial tension between two phases. The gravity effect was neglected because the gravitational force is negligible when the drop diameter is in the order of micrometers [41]. Capillary numbers of the continuous phase, *Ca*, represents the ratio of viscous force to interfacial tension and can be defined by,

(6) $$Ca=\frac{\eta_{\mathrm{CP}}V_{\mathrm{CP}}}{\sigma }$$

**Figure 5:**
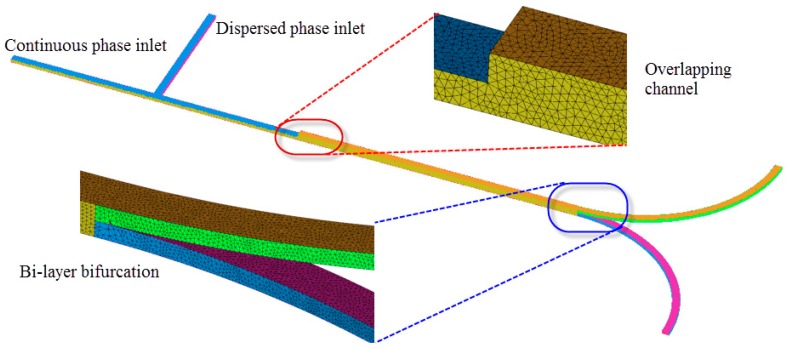
Tetrahedral grids of the bi-layer bifurcating microchannel.

**Figure 6:**
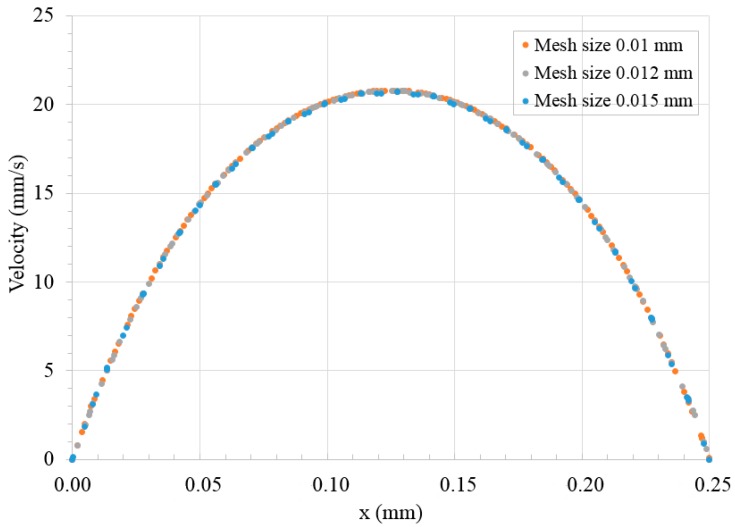
Velocity profile with various mesh sizes.

The governing equations were discretized to algebraic equations using a control-volume-based technique. The PISO (Pressure-Implicit with Splitting of Operators) algorithm was used in the transient calculations for droplet generation and fission. The momentum equation was discretized using second order upwind scheme, and the pressure interpolation was achieved by Pressure Staggering Option (PRESTO!) scheme. An iterative solver was deployed to solve the control-volume discretized equations. The iterative time step is 10^−6^ s and the solution converges when the residual is below a tolerance set as 1.0 × 10^−6^. No-slip condition was applied at the solid boundaries of the walls of the microchannel. Zero gauge pressure was applied at the outlet of the microdevice. The flow velocity was specified at each inlet of the microdevice. The numerical data were analyzed by the Ansys CFX-Post Processor 15.0. The simulations were performed using numerical grids composed of tetrahedral elements generated by ANSYS package ICEM CFD 15.0 (see Figure 5). The accuracy of the numerical model was validated by grid size sensitivity study to evaluate the effect of different tetrahedral grid sizes and ensure the simulation results are grid size independent. Comparison has been conducted for velocity profiles in widthwise direction along middle line of the same cross section located 1 mm upstream of bi-layer bifurcating junction. Three different mesh sizes, 0.01, 0.012 and 0.015 mm, have been used in the investigation with flow rate of 3 and 9 μL/min for dispersed and continuous phase, respectively. Good agreement of the parabolic velocity profile has been found among the three cases, as shown in Figure 6, demonstrating that the optimal mesh size is 0.012 mm. The mesh size of 0.01 mm is not used to save the computational cost.

## 4. Results and Discussion

**Figure 7:**
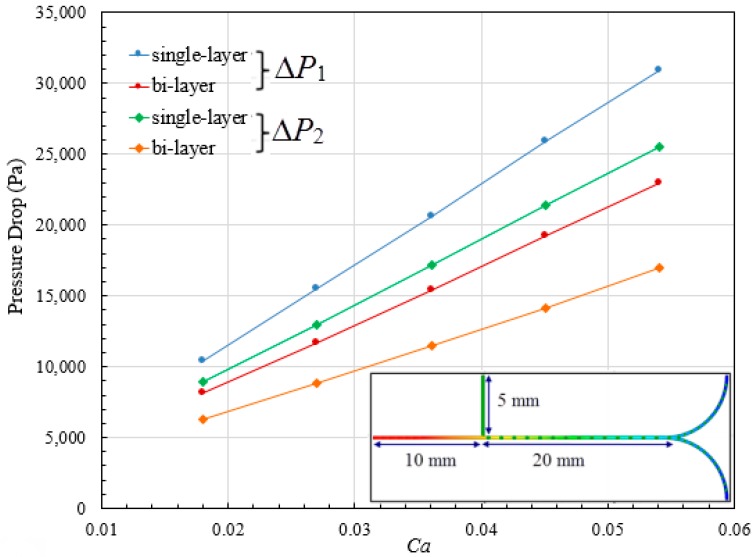
Pressure drop in bi-layer versus single-layer bifurcating microchannel at different Capillary numbers.

First, the pressure distribution in single-layer (see Figure 1c) versus bi-layer bifurcating microchannel (see Figure 1d) has been investigated with different Capillary numbers via control over the flow rate of continuous phase. The quantitative comparison results can be found in Figure 7. The pressure drop Δ*P*_1_ between continuous phase inlet and outlet, and the pressure drop Δ*P*_2_ between dispersed phase inlet and outlet have been calculated and plotted against Capillary number spanning a range from 0.018 to 0.054. Δ*P*_1_ is higher than Δ*P*_2_ for both microsystems, due to the longer distance from the continuous phase inlet to the outlet (see the inset in Figure 7), as well as the higher flow rate of the continuous phase. The total pressure drop increases as Capillary number increases because larger pressure gradient is required to overcome more pronounced viscous effect at higher flow rate. At the same Capillary number, higher pressure drop is demanded in single-layer microchannel, for example, Δ*P*_1_ is 11,683.91 Pa in bi-layer channel when *Ca* = 0.027, while it increases to 15,496.38 Pa in single-layer channel. 

**Figure 8:**
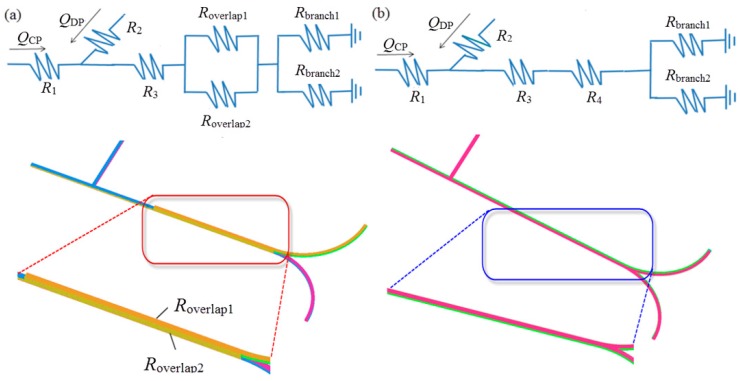
(**a**) Model of flow resistance and corresponding geometry of bi-layer microchannel; (**b**) model of flow resistance and corresponding geometry of single layer microchannel.

The variations in pressure drop for two types of microsystem can be explained using the flow resistance model. The bi-layer and single-layer microchannel structure are regarded as resistive circuit, as shown in Figure 8a,b, respectively, where *R* is the flow resistance of a single microchannel segment, and *Q* is the volumetric flow rate. In each segment with fixed flow resistance, higher-pressure drop results in higher flow rate. Compared to the single-phase flow system, the flow condition becomes complex due to the presence of interfaces in multiphase flow system. In this study, we simply assumed that the overall pressure drop is the sum of pressure drops derived from each microchannel segment. The pressure drop ΔP can be correlated with the volumetric flow rate *Q* of incompressible Newtonian fluid in a microchannel with rectangular cross-sections by [42],

(7) $$\Delta P=Q\times \frac{12\mu L}{w^{3}h}{\left[1-\frac{192w}{\pi^{5}h}\overset{\infty }{\underset{n=1,3,5}{\sum }}\frac{\tan h\left(\frac{n\pi h}{2w}\right)}{n^{5}}\right]}^{-1}$$

where *w* is th width of the channel, *h* is height of the channel, *L* is the length of the channel and can be estimated by *L* = *rθ* for curved microchannel. The geometric flow resistance can be represented by the second term on the right hand side of Equation (7). Flow resistance is therefore proportional to channel length, and higher flow resistance can arise from either increase of radius of curvature or included angle of channel, leading to higher pressure drop with the same flow rate. With the same volumetric flow rates at inlets, pressure drop is directly proportional to flow resistance, the value of which is larger in single-layer microstructure, because two lapped segments (see inset in Figure 8a) are arranged in parallel, resulting in relatively smaller flow resistance for bi-layer bifurcating microchannel.

**Figure 9:**
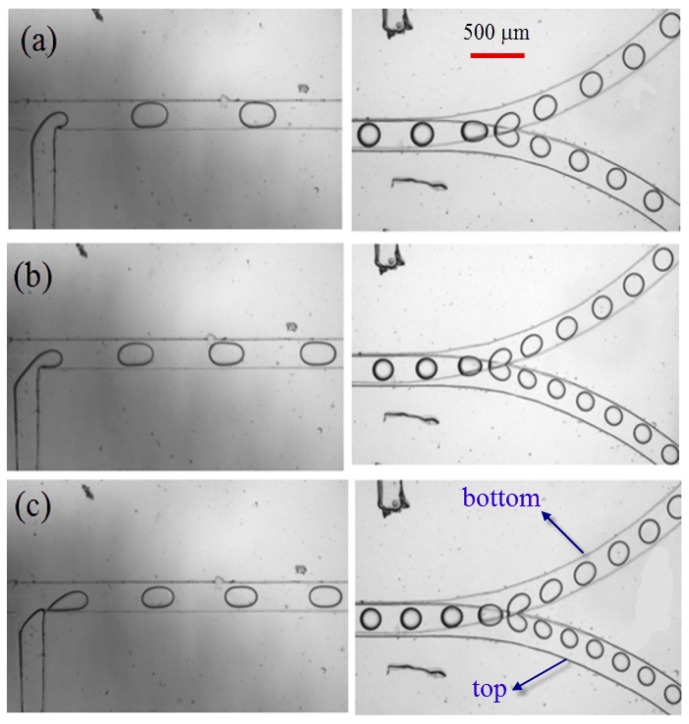
Droplet formation (left) and fission (right) in bi-layer bifurcating microchannel with dispersed phase flow rate of 2, 4 and 6 μL/min and continuous phase flow rate of 6, 12 and 18 μL/min in (**a**–**c**), respectively, the scale bar denotes 500 μm.

Emphasis will subsequently be given to droplet formation and fission in bi-layer bifurcating microchannel at various flow conditions by adjusting the flow rate of continuous phase and dispersed phase. The captured images from experimental recordings are shown in Figure 9. As the dispersed phase is purged out of the T-junction and progresses into the main channel, the shear force from continuous phase exerts on the dispersed phase thread which consequently becomes thinner. As viscous shear stress overcomes the interfacial tension, the dispersed phase directly breaks up into bullet-like shaped droplets, detaching from main thread at T-junction, and migrating further downstream along the overlapped main channel section which has height of 0.2 mm. The expansion of the channel height from 0.1 mm to 0.2 mm leads to reduced shear stress, making droplets adopt spherical shape as interfacial tension dominates over shear stress. The train of spherical droplets continues to move downstream to approach the bi-layer bifurcating junction, where two lobes are developed due to the established shear stress. The lobe is still attached to a mother droplet, while each partially blocks the branch curved channel, the pressure is built up to some extent that viscous shear stress is large enough to overcome the interfacial tension. Consequently, the lobes are detached from the mother droplet, forming two daughter droplets in top and bottom layer, respectively. It is of interest to note that the train of daughter droplets strikes against outer wall of channel as they migrate along curved bifurcating microchannel, because they are subject to centrifugal force which is exerted along radially outward direction from the center of curvature. 

**Figure 10:**
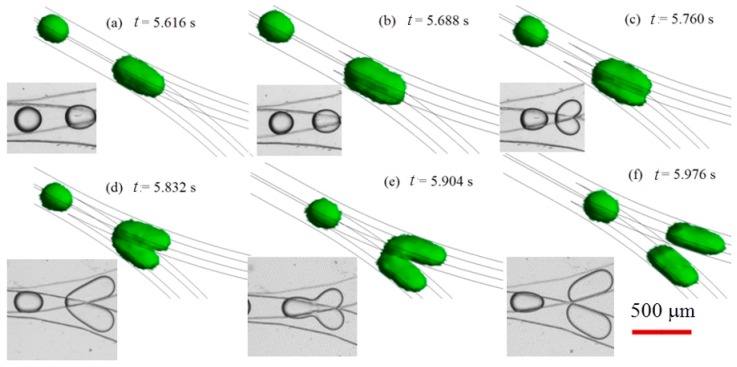
Time lapse images of droplet fission in bi-layer bifurcating microchannel from simulation and experiments when *Ca* = 0.054. The red scale bar denotes 500 μm.

**Figure 11:**
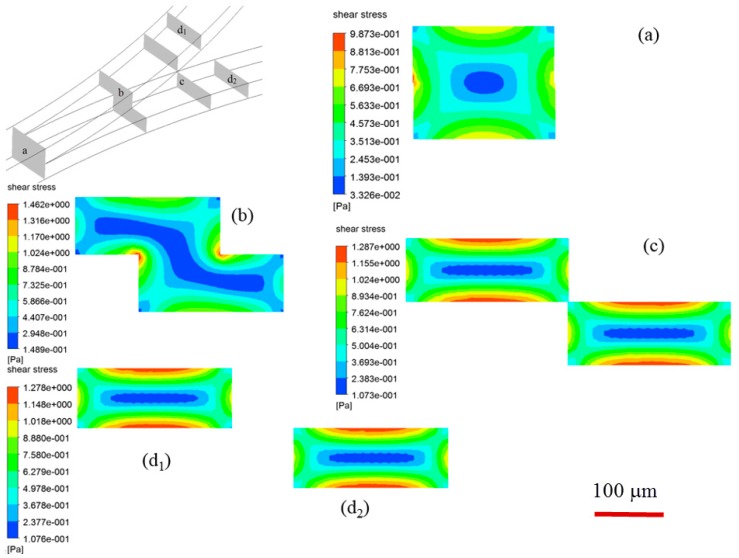
Shear stress distribution at various cross-sectional planes in microchannel, (**a**,**b**) locations upstream of bi-layer bifurcating junction; (**c**) location at singularity point; (**d**) location downstream of bi-layer bifurcating junction, subscripts 1–2 denotes top and bottom channel, respectively. The scale bar denotes 100 μm.

The experiments allow for observation of droplet formation and splitting in 2-D view. Numerical simulation has been conducted to supplementarily unveil the droplet fission associated breakup dynamics in 3-D perspective, and the temporal evolution of droplets with dispersed phase flow rate of 6 L/min and continuous phase flow rate of 18 μL/min is compared with experiments when *Ca* = 0.054 (see Figure 10). The interface between water and oil is represented by green color. As the spherical mother water-in-oil droplets migrate towards the singularity point of the bifurcating junction between two layers, there is significant droplet deformation, i.e., the droplet extends in stream-wise direction and the front part of droplet squeezes into the constriction of each branch channel due to the hydrodynamic pressure, the top and bottom parts of a mother droplet start to drain into upper and lower channel, respectively (see Figure 10 when *t* = 5.760 s). The two parts are dislodged and finally detached from each other (see Figure 10 when *t* = 5.976 s). The breakup of a mother droplet at bi-layer bifurcating junction is affected by shear stress distribution, as illustrated in Figure 11. Shear stress distribution contours at various cross-sectional planes indicate that the highest shear stress distributes in vicinity of wall boundary, whilst the lowest shear stress distributes in the center of cross section of microchannel. The applied shear stress on a droplet will facilitate fission in diagonal direction (see Figure 11b,c), leading to enhanced droplet fission efficiency. 

**Figure 12:**
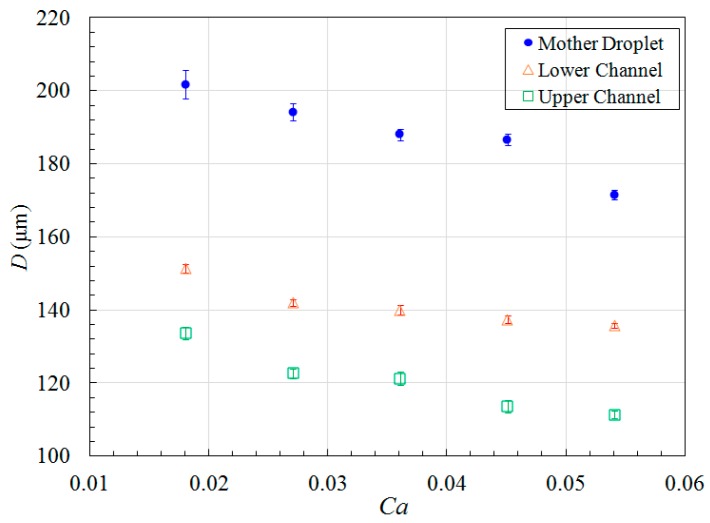
Average Feret’s diameter of droplets measured at various Capillary numbers during fission process in bi-layer bifurcating microchannel.

**Figure 13:**
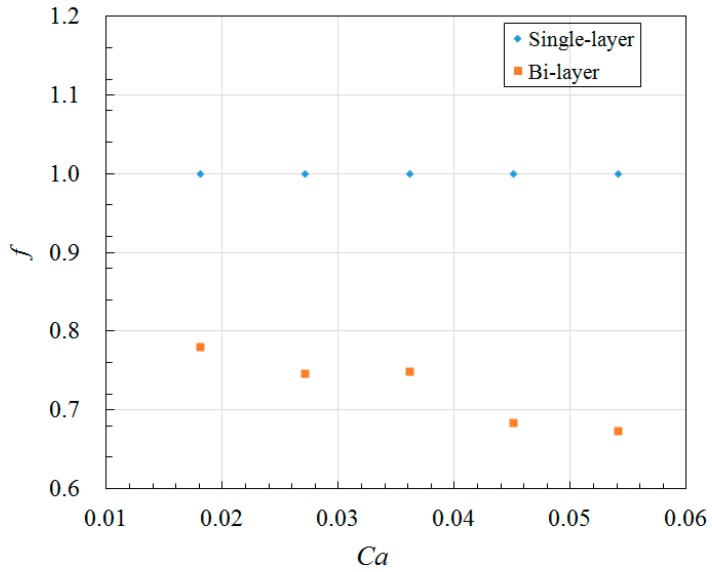
Volume ratio of daughter droplets in single-layer versus bi-layer bifurcating microchannel.

The droplet size in lower channel is larger than that in upper channel, indicating formation of unequally sized daughter droplets as shown in Figure 9, this is completely different from single-layer bifurcating microchannel where symmetric breakup of a mother droplet can be achieved [36]. The Feret’s diameter of mother droplets in main channel, as well as the Feret’s diameter of daughter droplets in lower and upper channels have been measured at different Capillary numbers, and the average size together with error bar is demonstrated in Figure 12. The small error bar indicates good monodispersity of droplet size distribution in each branch curved microchannel. Increased flow rate results in enhanced shear stress, leading to formation of droplets with smaller size. For example, the average Feret’s diameter of mother droplets is 201.7 μm at flow rate of 8 μL/min when *Ca* = 0.018, while it is reduced to 171.5 μm at flow rate of 24 μL/min when *Ca* = 0.054. Quantitative comparison of size between daughter droplets in different layers can be found from Figure 12 as well. It clearly shows that the average Feret’s diameter of daughter droplets in lower channel is larger than that of daughter droplets in upper channel. For instance, the average Feret’s diameter is 137.4 μm for daughter droplets in lower channel, while 113.6 μm for daughter droplets in upper channel at flow rate of 20 μL/min when *Ca* = 0.045. It indicates that asymmetric cutting of droplets using one bi-layer bifurcating junction can passively break up droplets into daughter droplets with two sizes, each of which has controlled monodispersity. Multiple sizes can be achieved with scale-up of the bi-layer bifurcating junctions. Assuming volume ratio of the daughter droplets generated by bifurcating junction is represented by *f*, the results of volume ratios at different Capillary numbers are shown in Figure 13, demonstrating that symmetric breakup of mother droplets can be achieved via single-layer bifurcating microchannel, whereas asymmetric breakup of mother droplets can be achieved via bi-layer bifurcating microchannel. The size variation increases with Capillary number for the latter case. 

**Figure 14:**
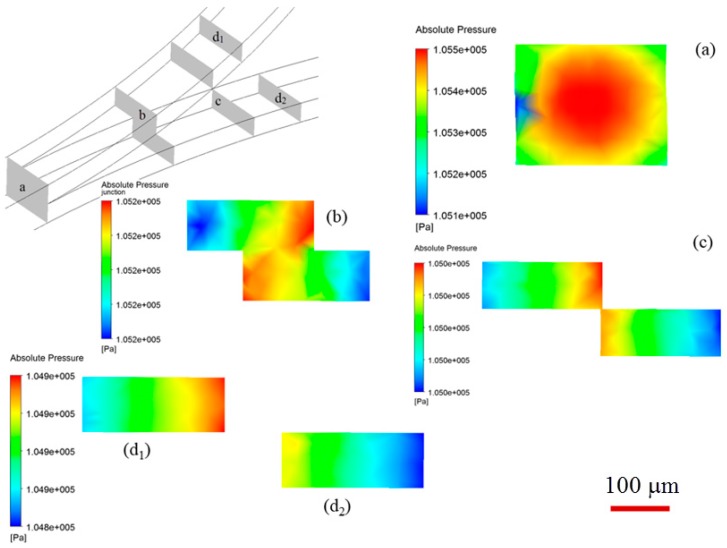
Pressure distribution at various cross-sectional planes in microchannel when the dispersed and continuous phase flow rate is 6 and 18 μL/min, respectively, (**a**,**b**) locations upstream of bi-layer bifurcating junction; (**c**) location at singularity point; (**d**) location downstream of bi-layer bifurcating junction, subscripts 1–2 denotes top and bottom channel, respectively. The scale bar denotes 100 μm.

**Figure 15:**
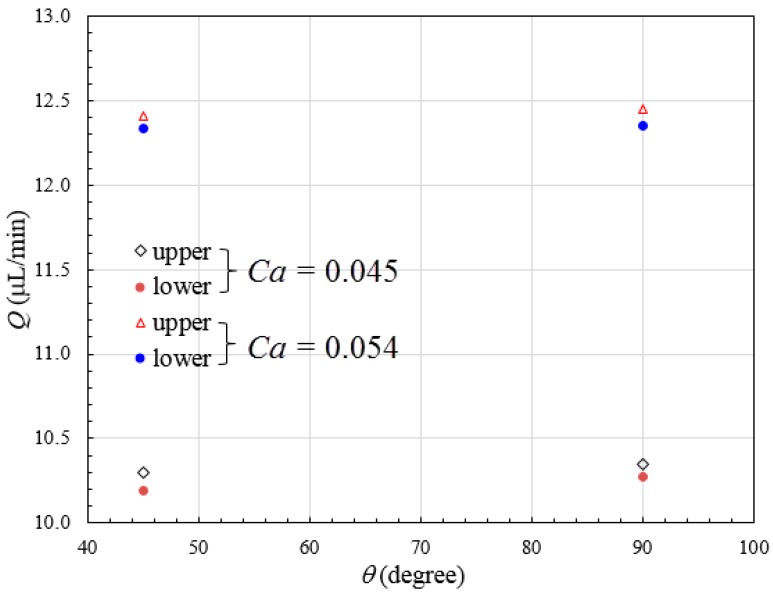
Flow rate distribution in upper and lower branch microchannels with different included angles and Capillary numbers. The dispersed and continuous phase flow rate is 5 and 15 μL/min, respectively when *Ca* = 0.045, and 6 and 18 μL/min, respectively when *Ca* = 0.054.

We attribute the droplet size variation to be related with the different pressure distribution in upper and lower channels. The presence of stepwise constriction leads to elevation head (see cross-section views in Figure 1d), the total pressure at entrance of upper channel will thus be larger than that at entrance of lower channel, assuming the sum of static pressure and dynamic pressure is the same for both channels. The total pressure drop in upper channel is higher than that in lower channel. In the present study, the upper and lower channels have the same included angle of 90° and radius of curvature of 5 mm, thus possessing the same geometric flow resistance (see Equation (7)). Higher flow rate can be expected in upper channel, leading to higher shear stress, and thus reduced droplet size. To verify the hypothesis, the pressure distribution across several cross-sectional planes along bi-layer microchannel at various positions is shown in Figure 14. Relatively higher pressure is distributed in upper channel. The unequal distribution of flow rate can be found from Figure 15 which shows numerically measured flow rate in upper and lower microchannels at different Capillary numbers with included angles of 45° and 90°, respectively. Flow rate increases with Capillary number, and flow rate in upper channel is larger than that in lower channel, irrespective of included angle. 

Finally, in order to fully exploit the potential of passive breakup by bi-layer bifurcating junction, we must elucidate the physics as for how the drop breakup occurs, and determine the conditions under which they split into two droplets. To this end, a simple analysis was made to determine the boundary between breakup and non-breakup regimes using a phase diagram. When a droplet of initial length *c*_0_ and diameter *D*_0_ breaks at the bi-layer bifurcation, one part of it with small diameter of *D*_1_ confined to the upper channel, while the other one with large diameter of *D*_2_ confined to the lower channel. Assuming that each part obtains a final length of *c*_1_ and *c*_2_, respectively. Mass conservation gives

(8) $$\frac{\pi }{4}D_{0}{}^{2}\times c_{0}=\frac{\pi }{4}D_{1}{}^{2}\times c_{1}+\frac{\pi }{4}D_{2}{}^{2}\times c_{2}$$

Since the volume ratio of the smaller daughter droplet to the larger one is *f*,

(9) $$D_{0}{}^{2}\times c_{0}=\left(1+f\right)\;\times D_{2}{}^{2}\times c_{2}$$

According to the Rayleigh–Plateau instability, a liquid column with circular cross-section reduces its surface area by breaking up when its length exceeds its circumference [43]. Therefore the elongated length of a droplet which is going to break up at least equals its circumference:

(10) $$c_{2}=\pi \times D_{2}$$

The critical capillary number for breakup *Ca*_cr_ is related to the initial and elongated lengths of the droplet according to Equation (11) [29],

(11) $$Ca_{\mathrm{cr}}\propto (c_{2}\;-\;c_{0}){}^{2}$$

**Figure 16:**
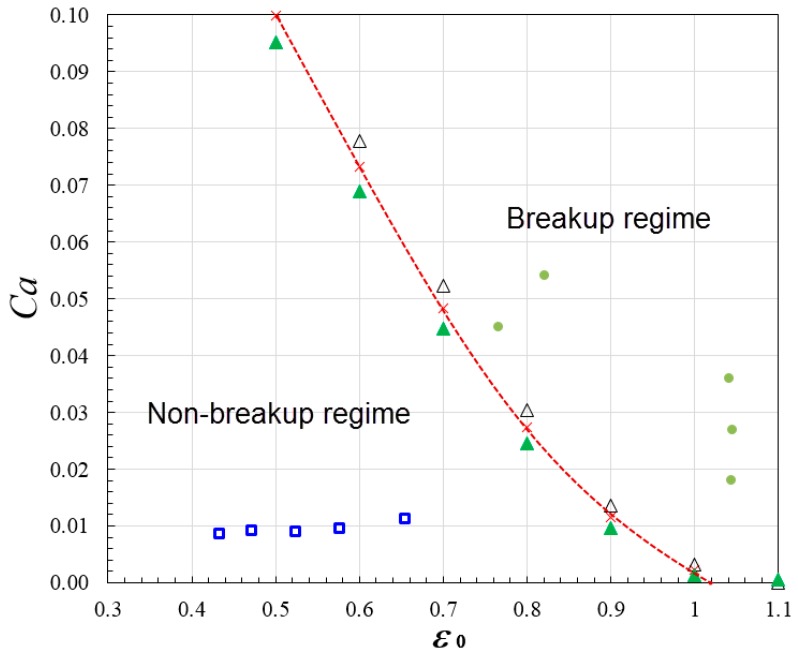
Phase diagram of droplet breakup at bi-layer bifurcating junction when fitting parameter *β* = 0.9, included angle *θ* = 45°, width *w* = 0.25 mm, height *h* = 0.1 mm and radius of curvature *r* = 5 mm. The breakup cases are represented by solid dot symbols and the non-breakup cases are represented by hollow square symbols. The two regimes are separated by a critical Capillary condition line obtained with droplet size ratio *f* = 0.73. The hollow and solid triangle symbols denote the critical Capillary number obtained when *f* = 0.68, and 0.78, respectively.

Combing Equations (9)–(11), we can obtain

(12) $$Ca_{\mathrm{cr}}=\beta \epsilon_{0}{}^{2}{\left[\epsilon_{0}{}^{-2/3}{\left(\frac{2}{1+f}\right)}^{1/3}-1\right]}^{2}$$

in which β is a parameter depending on the channel geometry of the bi-layer bifurcation, and $\epsilon_{0}$ is the droplet’s initial dimensionless length $\epsilon_{0}$ = $\frac{c_{0}}{\pi \times D_{0}}$. The phase diagram shows the Capillary number as a function of the dimensionless droplet initial length, the region of breakup represented by solid dot symbols and the region of non-breakup represented by hollow square symbols are demarcated by the critical Capillary condition line as depicted in Equation (12) with fitting parameter β = 0.9 specifically for the case when included angle is 45°, width is 0.25 mm and height is 0.1 mm for microchannel in each layer (see Figure 16). The droplet size of each daughter droplet was obtained from simulation by integration of the volume fraction, and the value of f can be obtained by finding the ratio of the smaller daughter droplet to the larger one. The value of ratio *f* ranges from 0.68 to 0.78 in the cases as depicted in Figure 16, an average value 0.73 was applied to create the critical Capillary condition line. The critical Capillary number obtained with droplet size ratio *f* = 0.68 and 0.78 respectively were also added in Figure 16, as represented by hollow and solid triangle symbols, respectively. It can be found the symbols are very close to the critical Capillary condition line created with *f* = 0.73. The phase diagram indicates that non-breakup occurs when *Ca* is below 0.012, the mother droplet migrates into the lower channel because of lower flow resistance and exits from the microdevice. It is also of interest to note that transition from breakup regime to non-breakup regime can be triggered by modifying the radius of curvature of bifurcating channels. For example, at the same flow rate when *Ca* = 0.054, when *r* = 5 mm for both channels, the droplet splits up into two daughter droplets after striking the stagnation point of bi-layer bifurcating junction (see Figure 10). In contrast, when the radius of curvature for upper channel is increased to 10 mm, due to significantly increased flow resistance (see Equation (7)), the droplets exit from port of lower channel without undergoing fission. We also found the droplets exiting port for non-breakup regime can be dynamically controlled by adjusting the height ratio of upper channel to lower channel. For example, the droplets exits from upper channel if height ratio is 3:2 while lower channel if height ratio is 2:3 when *Ca* = 0.008, *r* = 5 mm for both channels, *w* = 0.25 mm and *θ* = 45°, because reduction in channel height will lead to increase in flow resistance (see Equation (7)), preventing droplets from entering the channel. 

## 5. Conclusions

In this study, we have developed novel droplet fission approach using bi-layer bifurcating microchannel. The flow of emulsions and associated droplet breakup dynamics through bi-layer bifurcating junction have been investigated numerically and experimentally. Monodispersed daughter droplets are formed in upper and lower channel, while the average droplet size varies in different layers, arising from elevation loss formed at bi-layer bifurcating junction. Flow resistance can be tuned by controlling radius of curvature, and channel height ratio so that production of daughter droplets of distinct sizes can be achieved. Compared to bi-layer bifurcating microchannel at the same flow conditions, droplet fission is achieved at cost of high-pressure drop in single-layer bifurcating microchannel. The geometrically mediated droplet fission offers versatile control over droplet size distribution via asymmetric splitting. The microdevice is structurally simple and compact. Xurography process enables fabrication in facile way with low cost, obviating the need of expensive facilities such as clean room. The high scalability of the PDMS device can allow adding bifurcated junction per demand to create higher order emulsions, useful for synthesizing particles or capsules with customized inner compartments for encapsulation and release applications. Full exploitation of microfluidic production and control of emulsion droplets is severely limited if only a certain droplet size can be produced by single device. In contrast, our novel droplet fission platform allows for much better flexibility in forming droplets with distinct sizes while each size has good monodispersity. This would facilitate the use of droplets as micromixers or chemical reactors. Potential applications using the 3-D bi-layer bifurcating microstructure also include drug encapsulation with adjustable multiple dosages for drug delivery.

## Figures and Tables

**Table 1:** Property of the test fluids for droplet formation and fission.

Test Fluids	*η* (cP)	*ρ* (kg/m^3^)	*σ* (mN/m)
Cooking oil	64.3	908.7	14.26
Deionised (DI) Water	1.003	997	

## List of Symbols

Ca	Capillary number ($Ca=\frac{\eta_{\mathrm{CP}}V_{\mathrm{CP}}}{\sigma }$)
c	Length of droplet (m)
D	Feret’s diameter of droplet (m)
f	droplet volume ratio
*h*	Height of microchannel (m)
*L*	Length of microchannel (m)
*P*	Pressure (Pa)
*Q*	Flow rate (m^3^/s)
*R*	Flow resistance (kg/m^4^ s)
*r*	Radius of curvature (m)
*t*	Time variable (s)
*V*	Flow velocity (m/s)
*w*	Widthof microchannel (m)
x	Widthwise coordinate of microchannel (m)
*α*	Volume fraction
*β*	Correlation parameter
$\epsilon_{0}$	Initial dimensionless length of droplet
*σ*	Interfacial tension (N/m)
*θ*	Included angle of the channel (radian)
*ρ*	Density (kg/m^3^)
*η*	Dynamic viscosity (kg/m s)
